# Smoking Cessation and Incident Cardiovascular Disease

**DOI:** 10.1001/jamanetworkopen.2024.42639

**Published:** 2024-11-01

**Authors:** Jun Hwan Cho, Seung Yong Shin, Hoseob Kim, Mina Kim, Kyeongmin Byeon, Moonki Jung, Ki-Woon Kang, Wang-Soo Lee, Sang-Wook Kim, Gregory Y. H. Lip

**Affiliations:** 1 Division of Cardiology, Chung-Ang University Gwangmyeong Hospital, Gwangmyeong, Republic of Korea; 2 Division of Cardiology, Department of Internal Medicine, Korea University Ansan Hospital, Ansan, Republic of Korea; 3 Department of Data Science, Hanmi Pharm Co Ltd, Seoul, Republic of Korea; 4 Division of Cardiology, Department of Internal Medicine, Chung-Ang University Hospital College of Medicine, Seoul, Republic of Korea; 5 Liverpool Centre for Cardiovascular Science at University of Liverpool, Liverpool John Moores University and Liverpool Heart & Chest Hospital, Liverpool, United Kingdom; 6 Danish Center for Health Services Research, Department of Clinical Medicine, Aalborg University, Aalborg, Denmark; 7 Department of Internal Medicine, Chung-Ang University College of Medicine, Seoul, Republic of Korea; 8 Cardiovascular and Arrhythmia Center, Chung-Ang University Hospital, Seoul, Republic of Korea

## Abstract

**Importance:**

The association between smoking cessation and cardiovascular disease (CVD) risk in relation to cumulative smoking exposure remains poorly understood.

**Objective:**

To evaluate the associations among smoking cessation, lifetime smoking burden, and CVD risk according to the number of years elapsed after smoking cessation.

**Design, Setting, and Participants:**

This retrospective cohort study of the Korean National Health Insurance Service database investigated smoking duration and intensity between January 2006 and December 2008. Participants were categorized by self-reported smoking habits as current, ex-, or never-smokers. Smoking records were updated every 2 years until December 2019, with participants whose smoking status changed or whose smoking status was unclear excluded. Data analysis was performed between June and December 2022.

**Exposures:**

Time-updated self-reported smoking status, years since quitting, and cumulative smoking amount (pack-years [PY]).

**Main Outcomes and Measures:**

The primary outcome was incidence and hazard ratio of CVD (composite of cardiovascular death, myocardial infarction, stroke, and heart failure).

**Results:**

Overall, 5 391 231 participants (39.9% male; mean [SD] age, 45.8 [14.7] years; 853 756 [15.8%] current smokers, 104 604 [1.9%] ex-smokers, and 4 432 871 [82.2%] never smokers) were followed up for a mean (SD) of 4.2 (4.4) years. The median (IQR) baseline cumulative smoking amounts were 14.0 (7.5-20.0) PY in current smokers and 10.5 (5.3-20.0) PY in ex-smokers. The median (IQR) duration of smoking cessation was 4 (2-8) years for ex-smokers. Regardless of continued smoking, a dose-dependent association was evident between smoking and incident CVD. Compared with current smokers, ex-smokers with a lifetime smoking burden of less than 8 PY (light ex-smokers) experienced a significant reduction in CVD risk within 10 years of cessation, with a CVD risk similar to that of never-smokers. Conversely, ex-smokers with at least 8 PY (heavy ex-smokers) exhibited a slower decline in CVD risk than light ex-smokers, requiring more than 25 years for the residual CVD risk to disappear.

**Conclusions and Relevance:**

In this cohort study, smoking and CVD risk exhibited a dose-dependent association, with light ex-smokers having a CVD risk similar to that of never-smokers relatively soon after smoking cessation. For heavy ex-smokers, greater than 25 years might be required for the residual CVD risk to align with that of never-smokers.

## Key Points

**Question:**

How long does a person need to quit smoking to lower cardiovascular disease (CVD) risk?

**Findings:**

In this cohort study of extracted data from the Korean National Health Insurance Service database with more than 5 300 000 participants, ex-smokers who had accumulated less than 8 pack-years (PY) did not exhibit a significantly increased risk of CVD compared with never-smokers. However, for ex-smokers who had accrued at least 8 PY, more than 25 years were needed for the residual CVD risk of smoking to disappear.

**Meaning:**

These results suggest that ex-smokers with at least 8 PY should be considered at equivalent risk of CVD as current smokers, and management should be planned accordingly.

## Teaser

This cohort study investigates whether there are the associations among smoking cessation, lifetime smoking burden, and cardiovascular disease risk according to the number of years elapsed after smoking cessation.

## Introduction

Cardiovascular disease (CVD) is among the leading causes of death globally, and the health care and social burdens of CVD are continuously increasing.^1^ Smoking is a leading risk factor, and an important modifiable risk factor, for CVD.^1,2^ Smoking causes more than 8 million deaths worldwide each year,^3^ and among adults aged 30 to 44 years who died from ischemic heart disease, 38% of the deaths were attributed to smoking.^4^ As smoking cessation may reduce CVD risk,^5,6^ guidelines strongly recommend smoking cessation for primary and secondary prevention of CVD.^7,8^ Due to smoking cessation counseling, nicotine replacement therapy, and medication, along with improvements in cultural and social awareness, the smoking rate, which was 32.7% of global adult population in 2000, decreased to 22.3% in 2020.^4^ Similarly, in South Korea, the smoking rate decreased from 25.8% in 2012 to 20.6% in 2020.^9^ However, the potential benefits of smoking cessation on CVD risk modification have not yet been fully elucidated, especially in terms of lifetime smoking burden (pack-years [PY]) and temporal changes in CVD risk.

Indeed, the time elapsed after smoking cessation and temporal changes in CVD risk are not properly reflected in guidelines and contemporary clinical practice. For instance, the latest clinical CVD risk stratification tool does not properly estimate the potential CVD risk of ex-smokers.^10-12^ Indeed, the time elapsed after smoking cessation and lifetime smoking burden may affect CVD risk for a certain period. Therefore, this study aimed to evaluate the quantitative association between smoking cessation and subsequent CVD risk in association with the lifetime smoking burden and time elapsed after smoking cessation.

## Methods

For this retrospective cohort study, participants with complete smoking history were identified from 2006 to 2008, and were followed up until 2019 within the Korean National Health Insurance Service (NHIS) database. The NHIS is a mandatory health insurance program administered by the Korean government that covers approximately 50 million people (97% of the population). The Korean NHIS database includes inpatient and outpatient sociodemographic information, diseases diagnosed according to the *International Statistical Classification of Diseases and Related Health Problems, Tenth Revision (ICD-10)*, examinations, treatment with prescription dispensing records, and procedures.^13,14^ In addition, all beneficiaries of the NHIS aged at least 40 years receive free biennial health screening examinations, as provided by the Korean National Health Insurance Corporation. This health screening examination includes anthropometric measurements, blood pressure measurements, laboratory tests using blood and urine samples, chest radiography, and a self-reported standardized questionnaire on lifestyle behaviors, including smoking, alcohol intake, and physical activity.^14^ These data are linked to the Korean NHIS database, which is a National Health Screening database. Resident registration numbers are encrypted to protect individual information. All databases used in this study are available to researchers whose study protocols have been approved by the NHIS Data Sharing Service. This study was exempted from review by the institutional review board of Chung-Ang University and complied with the principles of the Declaration of Helsinki. This study followed the Strengthening the Reporting of Observational Studies in Epidemiology (STROBE) reporting guideline.

### Study Design and Study Population

**Figure 1.**
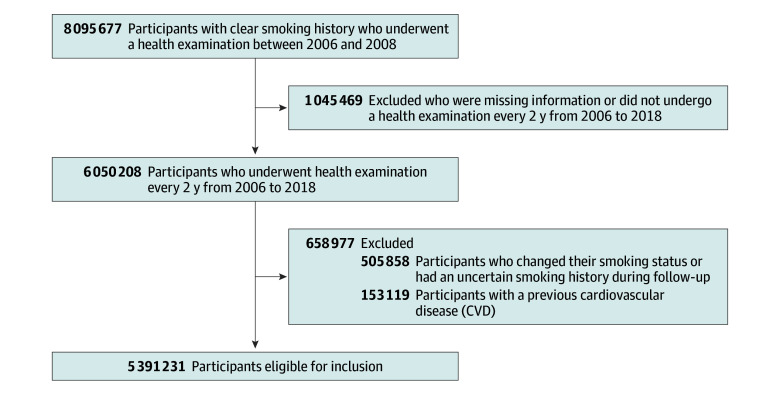
Study Flowchart

Overall, 8 095 677 participants underwent health screening between January 2006 and December 2008. To avoid misclassification bias and enhance the accuracy of smoking status, 2 045 469 participants who had an unclear smoking history and status at baseline or who did not undergo health screening every 2 years were excluded. Furthermore, 505 858 participants with an ambiguous smoking history or change in smoking status during the follow-up period and 153 119 with previous CVD were excluded. Therefore, current smokers included those who continued to smoke throughout follow-up period, ex-smokers included only those who quit smoking once and never smoked again, and all cases with unclear smoking records during the follow-up period were excluded from analysis. Consequently, 5 391 231 participants were included and followed-up from the index date until December 31, 2019. A detailed enrollment flowchart is shown in Figure 1.

### Quantification of Smoking Duration and Intensity

At the baseline health examination in 2006, participants were classified as current, ex-, or never-smokers using self-reported smoking habit data. For ex- and current smokers, we recorded the age at which the participant started smoking, number of cigarettes smoked per day (past or current), and age at quitting (ex-smokers only). These variables were used to calculate the cumulative PY at baseline for both current and ex-smokers, as well as years since quitting (YSQ). As PY and YSQ might affect CVD, they were updated at the biennial health screening to calculate cumulative smoking exposure and cessation variables.

### Defining Outcomes and Covariates

The primary outcome was the development of CVD, encompassing myocardial infarction, stroke, heart failure, and cardiovascular death. Myocardial infarction, stroke, and heart failure were identified according to *ICD-10* codes. The vital status, date of death, and cause of death were verified by linking records from the National Death Index using identification codes for each individual. Covariates included traditional CVD risk factors (hypertension, diabetes, and dyslipidemia), demographic data, and CVD-associated lifestyle behaviors, such as drinking and exercise. Comorbidities, including hypertension, diabetes, dyslipidemia, peripheral artery disease (PAD), kidney disease, liver disease, chronic obstructive pulmonary disease (COPD), and cancer, were identified using *ICD-10* codes. Definitions of the outcomes and comorbidities are listed in eTable 1 in Supplement 1.

Baseline variables such as body mass index (BMI), systolic and diastolic blood pressure, lipid profile (total cholesterol, triglyceride, high-density lipoprotein, and low-density lipoprotein), fasting glucose, and serum creatinine were collected from the health screening examination on the index date. The estimated glomerular filtration rate (eGFR) was calculated using the Modification of Diet in Renal Disease (MDRD) equation based on the measured serum creatinine levels. Data on alcohol consumption and regular exercise were collected using a self-reported structured questionnaire at the time of the health screening examination. Alcohol consumption was classified as nondrinker, mild-to-moderate drinker (>0 g/d to <30 g/d), or heavy drinker (≥30 g/d). Regular exercise was defined as performing at least 150 minutes per week of moderate physical activity, or at least 100 minutes per week of strenuous physical activity.^15^ South Koreans pay a fixed health insurance premium based on their income, and using the insurance premium data, we calculated the annual income levels of the participants and classified them into quintiles.

### Statistical Analysis

Categorical variables are presented as numbers and frequencies (percentages). Continuous variables are reported as the mean (SD) or median (IQR). We performed a Poisson regression analysis to determine the incidence rate of CVD. We confirmed a list of goodness-of-fit statistics, including the log-likelihood, Akaike information criterion (AIC), and Bayesian information criterion (BIC), to assess the fit of the model and adjusted for covariates such as age, sex, income levels, BMI, hypertension, diabetes, dyslipidemia, PAD, kidney disease, liver disease, COPD, cancer, heavy drinking, and regular exercise. Cox proportional hazards regression was used to estimate the adjusted hazard ratio (HR) of the primary and other outcomes according to smoking status. Before confirming the results, we assessed whether the proportional hazard assumptions were met. We adjusted for covariates. We also created a Cox model using restricted cubic splines with a greedy knot selection algorithm.^16^ Consequently, we finally chose 4 knots including the 5th, 30th, 80th, 85th percentiles (2, 8, 22, and 28 PY), based on the lowest AIC and BIC score. Significance was assessed using a 2-sided *P* < .05. SAS version 9.4. (SAS Institute) was used for the analyses from June to December 2022.

## Results

Among a total of 5 391 231 participants, 3 240 233 (60.1%) were women and 2 150 998 (39.9%) were male; 853 756 (15.8%) were current smokers, 104 604 (1.9%) were ex-smokers, and 4 432 871 (82.2%) were never smokers; the mean (SD) age was 45.8 (14.7) years. Most current smokers and ex-smokers were male (97% current smokers, 98.6% ex-smokers), whereas 27.5% of never-smokers were male. Participant characteristics at baseline are summarized in Table 1.

**Table 1.** Baseline Characteristics of Study Population

	Total population (n = 5 391 231)	Smoker			*P *value
		Current (n = 853 756)	Ex (n = 104 604)	Never (n = 4 432 871)	
Age at baseline, mean (SD), y	45.8 (14.7)	39.6 (11.7)	45.6 (11.3)	47.0 (15.0)	<.001
Sex, %					
Male	39.9	97.0	98.6	27.5	<.001
Female	60.1	3.0	1.4	72.5	
Income levels, quintile					
5th (highest)	1 100 204 (20.4)	157 568 (18.5)	14 426 (13.8)	928 210 (20.9)	<.001
4th	956 619 (17.7)	171 561 (20.1)	12 738 (12.2)	772 320 (17.4)	
3rd	988 910 (18.3)	196 225 (23.0)	18 400 (17.6)	774 285 (17.5)	
2nd	1 150 509 (21.3)	197 189 (23.1)	27 820 (26.6)	925 500 (20.9)	
1st (lowest)	1 194 989 (22.2)	131 213 (15.4)	31 220 (29.8)	1 032 556 (23.3)	
Anthropometric measurement					
BMI	23.4 (3.2)	23.8 (3.2)	24.4 (2.8)	23.3 (3.3)	<.001
Systolic blood pressure, mmHg	122.1 (16.2)	123.8 (14.2)	125.9 (14.4)	121.7 (16.5)	<.001
Diastolic blood pressure, mmHg	76.1 (10.5)	77.8 (9.9)	79.3 (9.9)	75.7 (10.6)	<.001
Baseline co-morbidity, %					
Hypertension	18.1	12.7	12.4	19.3	<.001
Diabetes	8.1	5.7	5.5	8.6	<.001
Dyslipidemia	16.3	11.3	11.3	17.4	<.001
Peripheral artery disease	4.9	3.4	3.4	5.2	<.001
Kidney disease	3.2	18.0	2.4	0.4	<.001
Liver disease	18.4	12.7	12.6	19.6	<.001
Chronic obstructive pulmonary disease	0.5	0.4	0.3	0.5	.97
Any malignant neoplasm	1.6	1.1	1.0	1.7	.42
Alcohol consumption, No. (%)^a^					
Nondrinker	3 217 320 (59.7)	184 612 (21.6)	24 576 (23.5)	3 008 132 (67.9)	<.001
Mild to moderate drinker (>0 g/d to <30 g/d)	1 835 263 (34.0)	511 107 (59.9)	62 894 (60.1)	1 261 262 (28.5)	
Heavy drinker (≥30 g/d)	326 006 (6.0)	156 459 (18.3%)	16 957 (16.2)	152 590 (3.4)	
Missing value	12 642 (0.2)	1578 (0.2)	177 (0.2)	10 887 (0.2)	
Regular exercise, No. (%)^b^	949 075 (17.6)	130 404 (15.3)	28 677 (27.4)	789 994 (17.8)	<.001
Laboratory findings					
Total cholesterol, mg/dL	192.4 (37.2)	191.7 (36.5)	196.1 (35.6)	192.5 (37.4)	<.001
Triglyceride, mg/dL	128.5 (96.9)	166.1 (36.5)	149.6 (105.2)	120.1 (89.2)	<.001
HDL cholesterol, mg/dL	56.8 (29.9)	52.9 (28.1)	53.7 (23.4)	57.8 (30.4)	<.001
LDL cholesterol, mg/dL	119.4 (159.4)	115.2 (158.9)	116.2 (79.2)	120.4 (161.1)	<.001
Fasting blood glucose, mg/dL	94.7 (23.5)	95.4 (25.1)	97.6 (23.5)	94.5 (23.2)	<.001
Estimated GFR, mL/min/1.73m^2^	88.1 (29.2)	95.9 (28.9)	90.1 (25.9)	86.4 (29.0)	<.001
Cigarettes per day, median (IQR)	NA	17 (10-20)	20 (10-20)	NA	NA
Cumulative smoking PY	NA	NA	NA	NA	NA
Median (IQR)	NA	14 (7.5-20)	10.5 (5.25-20)	NA	<.001
0 (never smoker)	4 432 871 (87.2)	NA	NA	4 432 871 (100)	
0-10 PY	303 530 (5.6)	264 195 (30.9)	39 335 (37.6)	NA	
10-20 PY	322 189 (6.0)	288 533 (33.8)	33 656 (32.2)	NA	
20-30 PY	177 162 (3.3)	159 921 (18.%)	17 241 (16.5)	NA	
≥30 PY	155 479 (2.9)	141 107 (16.5)	14 372 (13.7)	NA	
Years since quitting smoking, median (IQR)	NA	NA	4 (2-8)	NA	
Mean follow-up duration, y	4.2 (4.4)	5.0 (4.4)	6.1 (4.6)	4.0 (4.3)	<.001

Abbreviations: BMI, body mass index (calculated as weight in kilograms divided by height in meters squared); GFR, glomerular filtration rate; HDL, high-density lipoprotein; LDL, low-density lipoprotein; NA, not applicable; PY, pack-years.

SI conversion factors: To convert LDL to mmol/L, multiply by 0.0259; HDL to mmol/L, multiply by 0.0259; total cholesterol to mmol/L, multiply by 0.0259; blood glucose to mmol/L, multiply by 0.0555.

^a^ Self-reported consumption of at least 1 alcoholic beverage per week.

^b^ Self-reported questionnaire of regular exercise at least 150-minute/week.

Overall, the prevalence rates of hypertension, diabetes, and dyslipidemia were 18.1% (977 741 of 5 391 231), 8.1% (437 032 of 5 391 231), and 16.3% (880 344 of 5 391 231), respectively, and the never-smoker group had a significantly higher prevalence than the other 2 groups (Table 1). Among the study participants, 40.1% consumed alcohol at least once a week (current drinkers), and the proportion of heavy drinkers (≥30 g/d) was significantly higher among current smokers (18.3%; *P* < .001). In contrast, 67.9% of the never-smokers were nondrinkers.

The median (IQR) number of cigarettes smoked per day was 17.0 (10.0-20.0) for current smokers and 20.0 (10.0-20.0) for ex-smokers. The median (IQR) cumulative PY were 14.0 (7.5-20.0) for current smokers and 10.5 (5.3-20.0) for ex-smokers. Current and ex-smokers had similar cumulative PY distributions (eFigure 1 in Supplement 1). The median (IQR) YSQ for ex-smokers was 4 (2-8). The mean (SD) follow-up duration was 4.2 (4.4) years.

### CVD Risk by Smoking Status and Cumulative Smoking Amount

A total of 278 315 primary events occurred during the follow-up period. The crude event numbers, incidence, and HRs for CVD categorized by smoking cessation status and cumulative amount of smoking are presented in Table 2.

**Table 2.** Adjusted Risk of Primary Endpoint by Smoking Status and Amount

Smoking status	No.			Incidence rate per 1000 person-years (95% CI)		Hazard ratio	*P *value
	Person	Person-years	Event	Crude	Adjusted^a^		
Never	4 432 871	17 821 654	234 607	13.16 (13.11-13.22)	3.37 (3.28-3.48)	1 [Reference]	
Ex-smoker	104 604	633 798	7814	8.43 (8.35-8.52)	4.68 (4.54-4.84)	1.13 (1.10-1.15)	<.001
<10 PY	39 335	252 081	2008	4.58 (4.46-4.69)	3.87 (3.72-4.03)	1.01 (0.96-1.05)	.75
10-20 PY	33 656	211 956	2407	6.43 (6.30-6.56)	4.43 (4.27-4.59)	1.09 (1.05-1.14)	<.001
20-30 PY	17 241	100 758	1558	10.54 (10.31-10.76)	5.00 (4.82-5.19)	1.17 (1.11-1.23)	<.001
≥30 PY	14 372	69 003	1841	19.31 (18.96-12.61)	5.33 (5.15-5.53)	1.31 (1.25-1.37)	<.001
Current	853 756	4 257 153	35 894	12.33 (12.06-12.61)	6.03 (5.81-6.26)	1.22 (1.02-1.24)	<.001
<10 PY	264 195	1 319 044	6034	7.97 (7.63-8.32)	6.02 (5.71-6.36)	1.12 (1.09-1.14)	<.001
10-20 PY	288 533	1 540 489	9901	11.36 (10.91-11.82)	6.67 (6.34-7.02)	1.16 (1.13-1.19)	<.001
20-30 PY	159 921	800 907	8438	15.46 (14.71-16.25)	6.81 (6.42-7.23)	1.21 (1.18-1.24)	<.001
≥30 PY	141 107	596 713	11 521	26.68 (25.49-27.93)	6.90 (6.52-7.30)	1.36 (1.33-1.38)	<.001

Abbreviation: PY, pack-years.

^a^ Incidence rates and hazard ratios were adjusted for age, sex, income levels, body mass index, hypertension, diabetes, dyslipidemia, peripheral artery disease, kidney disease, liver disease, chronic obstructive pulmonary disease, cancer, heavy drinking, and regular exercise.

The incidence of CVD among ex-smokers and current smokers was significantly greater than that among never-smokers. The overall CVD incidences (per 1000 pack-years) among never smokers, ex-smokers, and current smokers were 3.37 (95% CI, 3.28-3.48), 4.68 (95% CI, 4.54-4.84), and 6.03 (95% CI, 5.81-6.26), respectively. The adjusted HRs of CVD risk for ex-smokers and current smokers compared with never-smokers were 1.13 (95% CI, 1.10-1.15; *P* < .001) and 1.22 (95% CI, 1.02-1.24; *P* < .001), respectively.

Each smoker group was stratified into 4 levels of lifetime smoking burden (0-10 PY, 10-20 PY, 20-30 PY, and ≥30 PY). In both the ex- and current-smoker groups, the incidence and risk of CVD increased with the cumulative lifetime smoking burden.

The dose-response association between PY and CVD among ex- and current smokers was positively correlated according to spline curves (eFigure 2 in Supplement 1). Ex-smokers with less than 8 PY (light ex-smokers) did not have a significantly greater risk of CVD than did never-smokers. Among current smokers, CVD risk increased linearly and rapidly up to 10 PY, with an HR of 1.01 (95% CI, 1.01-1.02) per person year up to 10 PY. However, the HR was similar from 10 to 20 PY and increased again after 20 PY.

According to the spline curve results, the HR of CVD in ex-smokers increased continuously without a plateau and it exceeded 1 at 8 PY (eFigure 2 in Supplement 1), whereas the HR of CVD in current smokers showed a plateau from 8 PY to 20 PY (eFigure 2 in Supplement 1), smoking amount could be classified as more or less than 8 PY. The associations between smoking amount and CVD risk in ex-smokers and current smokers were further analyzed (eTable 2 in Supplement 1). Compared with never smokers, light ex-smokers with less than 8 PY had a similar risk of CVD (HR, 1.02 [95% CI, 0.97-1.07]; *P* = .47), and ex-smokers with at least 8 PY (heavy ex-smokers) had a significantly greater risk of CVD (HR, 1.16 [95% CI, 1.13-1.19]; *P* < .001). For current smokers, participants with less than 8 PY had a CVD risk (HR) of 1.22 (95% CI, 1.18-1.26; *P* < .001); and participants with at least 8 PY had an HR of 1.22 (95% CI, 1.20-1.24; *P* < .001), respectively.

### CVD Risk by Smoking Cessation Duration

**Figure 2.**
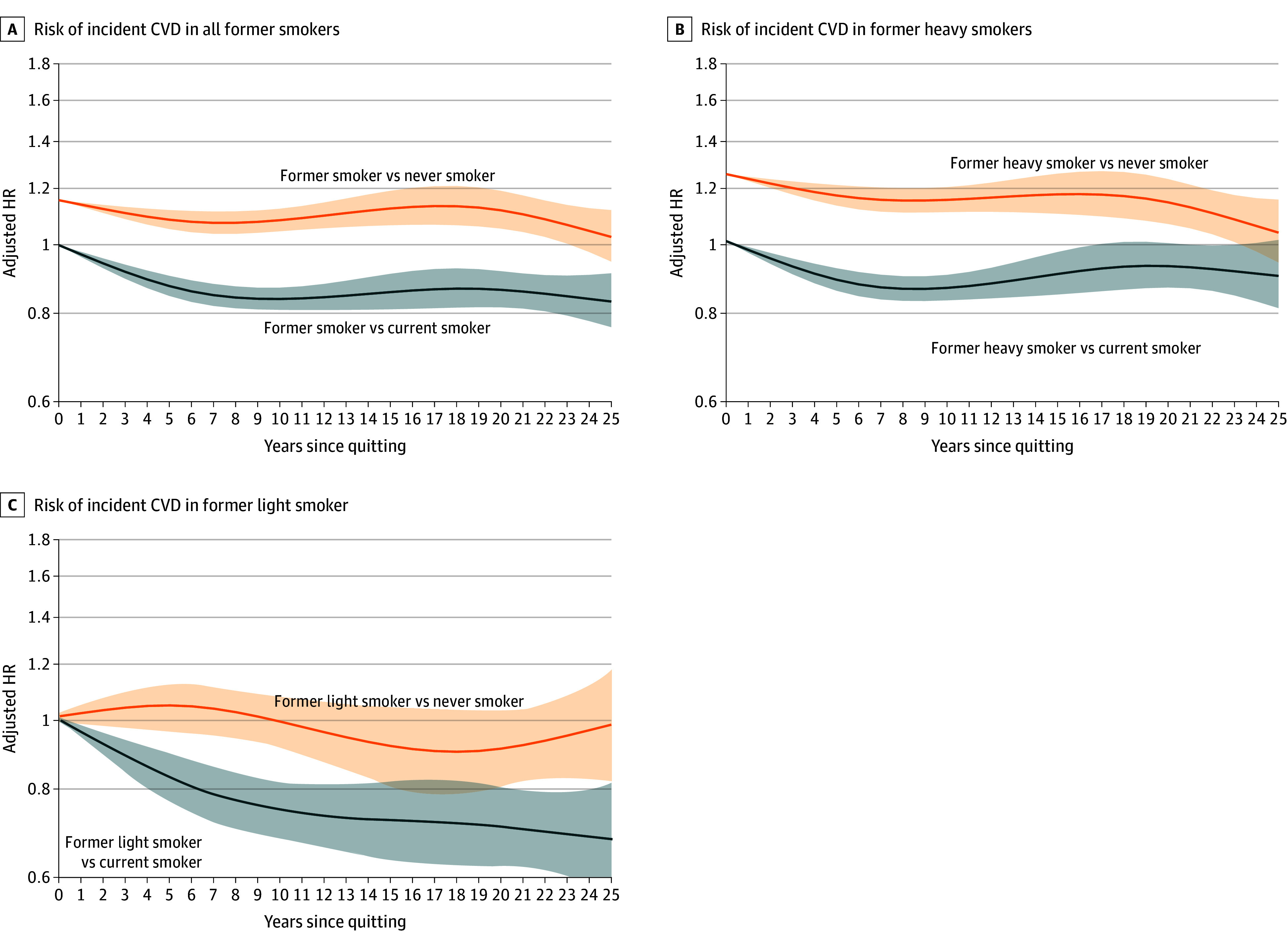
Risk of Incident Cardiovascular Disease in Ever and Never Smokers Heavy ex-smokers were ex-smokers with a lifetime smoking burden of ≥8 pack-years (PY); light ex-smokers were ex-smokers with a lifetime smoking burden of <8 PY. An adjusted HR greater than 1 indicates higher risk in the ex-smoker group. CVD indicates cardiovascular disease; HR, hazard ratio.

The association between elapsed time after smoking cessation and CVD risk is shown in Table 3 and Figure 2. CVD risk decreased with a longer duration of smoking cessation. Compared with that of current smokers, CVD risk among ex-smokers decreased relatively soon after quitting smoking (<10 years).

**Table 3.** Multivariable-Adjusted Risk of Incident CVD by Smoking Status and YSQ

	No.			Smokers			
	Person	Person-year	Event	Ex vs current		Ex vs never	
				HR (95% CI)^a^	*P*-value	HR (95% CI)^a^	*P *value
Current smokers	853 756	4 257 153	35 894	1 [Reference]		1.22 (1.20-1.33)	<.001
YSQ <5	53 347	330 921	3484	0.96 (0.92-0.99)	.001	1.17 (1.13-1.21)	<.001
5 ≤YSQ <10	29 606	181 315	2216	0.91 (0.87-0.95)	<.001	1.11 (1.06-1.15)	<.001
10 ≤YSQ <15	10 656	62 440	913	0.91 (0.85-0.97)	.006	1.11 (1.04-1.19)	.002
1 5≤YSQ <20	5330	30 617	502	0.90 (0.83-0.99)	.03	1.10 (1.01-1.21)	.03
20 ≤YSQ <25	2925	15 102	336	0.96 (0.86-1.07)	.45	1.17 (1.05-1.30)	.004
25 ≤YSQ	2740	13 403	363	0.91 (0.82-1.01)	.07	1.11 (1.00-1.23)	.05
Never smoker	4 432 871	17 821 654	234 607	0.82 (0.81-0.83)	<.001	1 [Reference]	

Abbreviations: CVD, cardiovascular disease; HR, hazard ratio; YSQ, years since quitting.

^a^ Hazard ratios were adjusted for age, sex, income levels, body mass index, hypertension, diabetes, dyslipidemia, peripheral artery disease, kidney disease, liver disease, chronic obstructive pulmonary disease, cancer, heavy drinking, and regular exercise.

Using current smokers as the reference group, ex-smokers had significantly lower risk of CVD within 5 years of quitting (HR, 0.96 [95% CI, 0.92–0.99]; *P* = .001) and 5 to 10 years after quitting smoking (HR, 0.91 [95% CI, 0.87–0.95]; *P* < .001). Compared with never-smokers, a significantly greater CVD risk persisted for more than 20 years after smoking cessation. As shown in Figure 2, the crossing point at which the 95% CI for CVD risk between ex- and never-smokers consistently contained null values of 1 was 23 years after cessation.

For light ex-smokers, smoking cessation was associated with a rapid decline in CVD risk compared with current smokers, and was significantly lower within 20 years of smoking cessation (YSQ <5 years: HR, 0.91 [95% CI, 0.83-0.99]; 5 ≤YSQ <10 years: HR, 0.85 [95% CI, 0.77-0.94]; 10 ≤YSQ <15 years: HR, 0.85 [95% CI, 0.74-0.97]; 15 ≤YSQ <20 years: HR, 0.74 [95% CI, 0.61-0.89]) There was no significant difference in CVD risk between light ex-smokers and never-smokers (eTable 3 in Supplement 1; Figure 2).

Even for heavy ex-smokers, CVD risk was significantly greater than that for never-smokers up to 25 YSQ. CVD risk was statistically similar between heavy ex-smokers and current smokers until 20 YSQ (YSQ <5 years: HR, 0.97 [95% CI, 0.95-0.99]; 5 ≤YSQ <10 years: HR, 0.92 [95% CI, 0.88-0.97]). Compared with that of never-smokers, the residual CVD risk of heavy ex-smokers persisted for up to 25 years (YSQ ≥25 years: HR, 1.18 [95% CI, 0.94-1.43]) (eTable 3 in Supplement 1; Figure 2).

## Discussion

In this cohort study, our principal results were as follows: (1) smoking and CVD risk exhibited a dose-response association regardless of smoking cessation; (2) for all ex-smokers, smoking cessation was associated with significantly reduced CVD risk, but the subsequent CVD risk of ex-smokers was determined by their cumulative amount of smoking and their YSQ; and (3) the amplitude of CVD risk reduction after smoking cessation varied depending on the cumulative amount of previous smoking. For heavy ex-smokers, at least 25 years had to elapse for their subsequent CVD risk to align with that of never-smokers; however, for light ex-smokers, the association of smoking cessation with lower CVD risk was immediately apparent. The subsequent CVD risk within 5 YSQ for light ex-smokers was significantly lower than that for current smokers and similar to that of never-smokers.

These results have important implications for clinical practice and public health. Regardless of smoking cessation status, smoking and CVD risk exhibit a clear dose-response association, emphasizing the importance of preventing smoking initiation altogether. For people who start smoking, if their cumulative smoking amount does not exceed a certain threshold or the so-called point of no return, 8 PY in this study, they may quit smoking with marked clinical improvements expected soon after quitting. Hence, this study’s results suggest that successful smoking cessation before reaching 8 PY promises considerable public health benefits.

Conversely, once the cumulative smoking amount surpasses 8 PY, even if smoking ceases, the residual CVD risk mirrors that of current smokers. Management should be planned according to the residual CVD risk equivalent to that of current smokers. Nevertheless, the residual risk associated with smoking can be nullified by sustained cessation over a sufficiently long period (≥25 YSQ), emphasizing the importance of long-lasting, permanent smoking cessation in these patients.

To the best of our knowledge, this is the largest and most comprehensive study on the association between the cumulative amount of smoking, lifetime smoking burden, elapsed time after smoking cessation, and CVD risk. Notable strengths include the use of a large database of participants and well-refined data. The exclusion of individuals with preexisting CVD at baseline enabled a focused analysis of de novo CVD incidence, which is mainly associated with smoking. More than 5 300 000 participants were analyzed and smoking and smoking cessation status were regularly checked during the follow-up period from 2006 to 2019, confirmed again using self-reported smoking ascertainment questionnaires, and the smoking status and amount of smoking were regularly updated.

### Dose-Response Associations Between Smoking and CVD Risk

Smoking causes oxidative stress, endothelial dysfunction, inflammation, and lipid modification in the long term, which promotes atherosclerosis and leads to CVD.^17^ Recent studies have demonstrated that smokers have more severe coronary plaque and coronary heart disease than never smokers.^18,19^ This study reaffirms the dose-response association between smoking and CVD and the cardiovascular benefits of smoking cessation, which were previously shown in other studies.^6,17,20,21^ This dose-response association was valid for current and ex-smokers. In our study, current smokers with 30 PY had a 2-fold greater risk of CVD than did never-smokers. However, ex-smokers with less than 10 PY did not have an increased CVD risk compared with never-smokers. The threshold of smoking that increased CVD risk in ex-smokers was 8 PY. Thus, the potential benefit of smoking cessation is more obvious when the cumulative amount of smoking (ie, lifetime smoking burden) is less than 8 PY, emphasizing the importance of earlier smoking cessation.

### Duration of Smoking Cessation and Subsequent CVD Risk

The reduction in CVD risk over time after smoking cessation has not been thoroughly investigated in prior studies. In this study, more than 20 years of smoking cessation were needed for the CVD risk of heavy ex-smokers to be comparable with that of never-smokers. These findings are broadly consistent with those of the British Regional Heart Study,^22^ in which the risk of myocardial infarction persisted at more than 20 YSQ. According to the results from the Framingham Heart Study, which included an original cohort and an offspring cohort of 8700 participants, smoking cessation in heavy ex-smokers (≥20 PY) significantly reduced CVD risk within 5 years compared with that in current smokers, but it took more than 15 years for CVD risk to be comparable with that of never-smokers.^20^ In contrast to the afovementioned studies, some studies^23,24^ found that the residual CVD risk after smoking cessation decreased if YSQ was less than 5 years, perhaps reflecting that the residual CVD risk was not high because most of the participants were light smokers.

In this study, light ex-smokers with less than 8 PY achieved a CVD risk similar to that of never smokers soon after quitting. However, heavy ex-smokers with at least 8 PY achieved a significantly lower CVD risk than current smokers within 5 YSQ, but it took a very long time for their CVD risk to become similar to that of never-smokers. In other words, the amount of smoking before quitting and the duration of smoking cessation are both important factors for subsequent CVD risk.

Widely adopted risk stratification schemes, such as the SCORE2 and Atherosclerotic Cardiovascular Disease risk calculators, tend to assume that ex-smokers have CVD risk that is similar with that of never-smokers, regardless of the cumulative smoking amount or the elapsed time after smoking cessation. Consequently, the residual CVD risk of ex-smokers may be underestimated by such simple estimations.^7,8^ Based on our findings, the residual CVD risk of ex-smokers should be quantitatively estimated based on the cumulative smoking amount and elapsed time after smoking cessation.

### Limitations

This study had limitations. First, this was an observational, retrospective cohort study using a nationwide claims database, and we could not infer causality. In addition, although covariates that may affect clinical outcomes have been considered and carefully adjusted for, confounding factors are likely to remain. However, considering the ethical issues of lifestyle modification studies (eg, randomizing participants to keep or stop smoking), randomized clinical trials are unlikely to support this hypothesis. In addition, given that the study relied on claims-based data, there may be potential errors in disease diagnosis due to inaccuracies in coding. To prevent such issues, we used diagnostic definitions that have been thoroughly validated in prior studies involving the Korean NHIS cohorts.^13-15,25^ Second, given the retrospective nature of this study, the amount of smoking was based on self-reported questionnaires and may differ from actual smoking. Objective measurements such as a urine cotinine test or exhaled carbon monoxide levels would be ideal,^26^ but information on environmental tobacco smoke exposure and the use of other types of tobacco was not available and was not included in this study. Third, although this study included a large population, only Asians of a single nationality were included, which should be considered when generalizing the findings. Fourth, the impact of smoking may differ by sex. Less than 5% of the current smokers and former smokers included in the analysis were women, so it is important to note that there are limitations in generalizing this study results to the female population. Therefore, additional research is needed to identify potential sex differences in the effects of smoking cessation.

## Conclusions

This cohort study found that smoking and CVD risk had a dose-dependent association, and light ex-smokers had a CVD risk similar with that of never-smokers relatively soon after smoking cessation. For heavy ex-smokers, at least 25 years had to elapse for the residual CVD risk of smoking to become similar to that of never-smokers. Thus, heavy ex-smokers should be considered to have a CVD risk equivalent to that of patients who continue to smoke, and management should be planned accordingly.

## Notes

Supplement 1. **eTable 1.** Definitions of Covariates **eTable 2.** Adjusted Risk of Primary Endpoint by Smoking Status and Amount **eTable 3.** Multivariable-Adjusted Risk of Incident CVD by Smoking Status and Years Since Quitting (YSQ) Smoking **eFigure 1.** Cumulative Pack-Years Among Current and Ex-Smokers **eFigure 2.** The Relationship Between Pack-Years and The Hazard Ratio of CVD in Ex-Smoker and Current Smoker

Supplement 2.
Data Sharing Statement
