# Supplementary material for: Smoking Cessation and Incident Cardiovascular Disease
Source: JAMA Netw Open. 2024 Nov 1;7(11):e2442639. doi: 10.1001/jamanetworkopen.2024.42639 (PMC11530932; doi:10.1001/jamanetworkopen.2024.42639)
Supplement: Supplement 1. — eTable 1. Definitions of Covariates eTable 2. Adjusted Risk of Primary Endpoint by Smoking Status and Amount eTable 3. Multivariable-Adjusted Risk of Incident CVD by Smoking Status and Years Since Quitting (YSQ) Smoking eFigure 1. Cumulative Pack-Years Among Current and Ex-Smokers eFigure 2. The Relationship Between Pack-Years and The Hazard Ratio of CVD in Ex-Smoker and Current Smoker [file jamanetwopen-e2442639-s001.pdf]

Supplemental Online Content

Cho JH, Shin SY, Kim H et al. Smoking cessation and incident cardiovascular disease. *JAMA Netw Open*. Published November 01, 2024. doi:10.1001/jamanetworkopen.2024.42639

eTable 1. Definitions of Covariates

eTable 2. Adjusted Risk of primary endpoint by smoking status and amount

eTable 3. Multivariable-Adjusted Risk of Incident CVD by smoking status and years since quitting (YSQ) smoking

eFigure 1. Cumulative Pack-Years Among Current and Ex-Smokers

eFigure 2. The Relationship Between Pack-Years and The Hazard Ratio of CVD in Ex-smoker and Current smoker

This supplemental material has been provided by the authors to give readers additional information about their work.

eTable 1. Definitions of Covariates

Diagnosis	ICD 10-CM code and definition	Diagnostic definition
**Clinical outcomes**		
Myocardial infarction	I21, I22	Admission or outpatient department≥1
Stroke	I60-I64	Primary diagnosis, admission≥1 (≥3 days) and brain imaging (CT or MRI) ≥1
Heart failure	I50	Admission or outpatient department≥1
**Comorbidities**		
Hypertension	I10-I13, I15; or minimum 1 prescription of antihypertensive drug (thiazide, loop diuretics, aldosterone antagonist, alpha-/beta-blocker, calcium-channel blocker, angiotensin-converting enzyme inhibitor, angiotensin II receptor blocker).<br>or systolic/diastolic blood pressure ≥ 140/90 mmHg	Admission≥1 or outpatient department≥2<br>Based on health examination
Diabetes mellitus	E11-E14; or minimum 1 prescription of antidiabetic drugs (sulfonylureas, metformin, meglitinides, thiazolidinediones, dipeptidyl peptidase-4 inhibitors, α-glucosidase inhibitors, SGLT2-inhibitor, GLP-1 agonist, and insulin).<br>or fasting glucose level ≥ 126 mg/dL	Admission≥1 or outpatient department≥2<br>Based on health examination
Dyslipidemia	E78; or minimum 1 prescription of lipid-lowering medication (statin, ezetimibe, fenofibrate)<br>or total cholesterol ≥ 240 mg/dL	Admission or outpatient department≥1<br>Based on health examination
Peripheral artery disease	I70, I73	Admission or outpatient department≥2

© 2024 Cho JH et al. *JAMA Network Open.*

Renal disease	I13.1, N03, N05, N10-N19, Z49, Z94.0, Z99.2	Admission or outpatient department≥1
Liver disease	K70, K72-K76, K71.3-K71.7	Admission or outpatient department≥1
Chronic obstructive pulmonary disease	J41-44	Admission≥1
Any malignancy	C00-97 and RID code (V193)	Admission or outpatient department≥1
**Health exam questionnaire**		
Alcohol consumption	Mild to moderate drinker: alcohol consumption > 0 g/day to <30g/day<br>Heavy drinker: alcohol consumption ≥30 g/day	Based on health examination
Regular exercise	Performing ≥150 min/week of moderate physical activity, or ≥100 min/week of strenuous physical activity	Based on health examination

© 2024 Cho JH et al. *JAMA Network Open.*

eTable 2. Adjusted Risk of primary endpoint by smoking status and amount

Smoking status	Person No.	Person-Years, No.	Event	Incidence Rate (crude) per 1000 person-years (95% CI)	Incidence Rate (adjusted)[a] per 1000 person-years (95% CI)	Hazard Ratio (95% CI)	*P*-value
**Never**	4,432,871	17,821,654	234,607	13.329 (13.111 – 13.218)	3.374 (3.275 – 3.476)	1 [Reference]	
**Former**							
< 8 PY	30,663	194,711	1,540	4.721 (4.579 – 4.867)	3.819 (3.659 – 3.986)	1.019 (0.968 – 1.073)	0.466
≥ 8 PY	73,941	439,087	6,274	9.390 (9.287 – 9.494)	4.839 (4.687 – 4.996)	1.156 (1.126 – 1.187)	<0.001
**Current**							
< 8 PY	183,408	873,954	4,126	7.909 (7.524 – 8.314)	5.532 (5.218 – 5.865)	1.215 (1.177 – 1.255)	<0.001
≥ 8 PY	670,348	3,383,199	31,768	14.289 (13.940 – 14.647)	6.189 (5.952 – 6.435)	1.219 (1.202 – 1.237)	<0.001

PY, pack-years

[a] Incidence rates and hazard ratios are adjusted for age, sex, income levels, body mass index, hypertension, diabetes mellitus, dyslipidemia, peripheral artery disease, renal disease, liver disease, COPD, cancer, heavy drinking, regular exercise

© 2024 Cho JH et al. *JAMA Network Open.*

eTable 3. Multivariable-Adjusted Risk of Incident CVD by smoking status and years since quitting (YSQ) smoking

1) Comparison among never smokers, current and ex-smokers with less than 8 pack-years (< 8 PY)

	Number	Person-year, No.	Event, No.	Former vs. Current smokers		Former vs. Never smokers	
				Hazard Ratio (95% CI)	*P*-value	Hazard Ratio (95% CI)	*P*-value
**Current smokers**	183,408	873,954	4,126	1 (Reference)		1.193 (1.155 – 1.233)	<0.001
**YSQ < 5**	15,188	97,138	613	0.905 (0.831 – 0.985)	0.022	1.080 (0.996 – 1.170)	0.061[a]
**5 ≤ YSQ < 10**	8,296	53,354	410	0.852 (0.770 – 0.943)	0.002	1.017 (0.922 – 1.121)	0.740
**10 ≤ YSQ < 15**	3,338	21,546	208	0.846 (0.736 – 0.973)	0.019	1.010 (0.881 – 1.158)	0.891
**15 ≤ YSQ < 20**	1,795	11,376	115	0.740 (0.614 – 0.893)	0.002	0.883 (0.734 – 1.063)	0.190
**20 ≤ YSQ < 25**	1,083	6,246	87	0.819 (0.662 – 1.013)	0.066	0.978 (0.792 – 1.206)	0.832
**25 ≤ YSQ**	963	5,051	107	0.820 (0.674 – 0.997)	0.047	0.979 (0.807 – 1.188)	0.828
**Never smoker**	4,432,871	17,821,654	234,607	0.838 (0.811 – 0.866)	<0.001	1 (Reference)	

2) Comparison among never smokers, current and ex-smokers with 8 PY or more (≥ 8 PY)

	Number	Person-year, No.	Event, No.	Former vs. Current smokers		Former vs. Never smokers	
				Hazard Ratio (95% CI)	*P*-value	Hazard Ratio (95% CI)	*P*-value
**Current smokers**	670,348	3,383,199	31,768	1 (Reference)		1.216 (1.198 – 1.233)	<0.001
**YSQ < 5**	38,159	233,783	2,871	0.968 (0.947 – 0.989)	0.013	1.177 (1.134 – 1.223)	<0.001
**5 ≤ YSQ < 10**	21,310	127,961	1,806	0.922 (0.879 – 0.968)	0.001	1.121 (1.070 – 1.176)	<0.001
**10 ≤ YSQ < 15**	7,318	40,894	705	0.938 (0.880 – 0.996)	0.045	1.140 (1.058 – 1.228)	<0.001
**15 ≤ YSQ < 20**	3,535	19,241	387	0.974 (0.881 – 1.077)	0.610	1.184 (1.071 – 1.309)	<0.001
**20 ≤ YSQ < 25**	1,842	8,856	249	1.034 (0.912 – 1.172)	0.605	1.257 (1.108 – 1.425)	<0.001
**25 ≤ YSQ**	1,777	8,352	256	0.969 (0.856 – 1.097)	0.615	1.178 (0.940 – 1.433)	0.813[b]
**Never smoker**	4,432,871	17,821,654	234,607	0.823 (0.811 – 0.834)	<0.001	1 (Reference)	

© 2024 Cho JH et al. *JAMA Network Open.*

* Hazard ratios are adjusted for age, sex, income levels, body mass index, hypertension, diabetes mellitus, dyslipidemia, peripheral artery disease, renal disease, liver disease, COPD, cancer, heavy drinking, regular exercise

[a,b] Point the statistical significance of CVD risk difference between former smokers and never smoker disappeared

© 2024 Cho JH et al. *JAMA Network Open.*

eFigure 1. Cumulative Pack-Years Among Current and Ex-Smokers

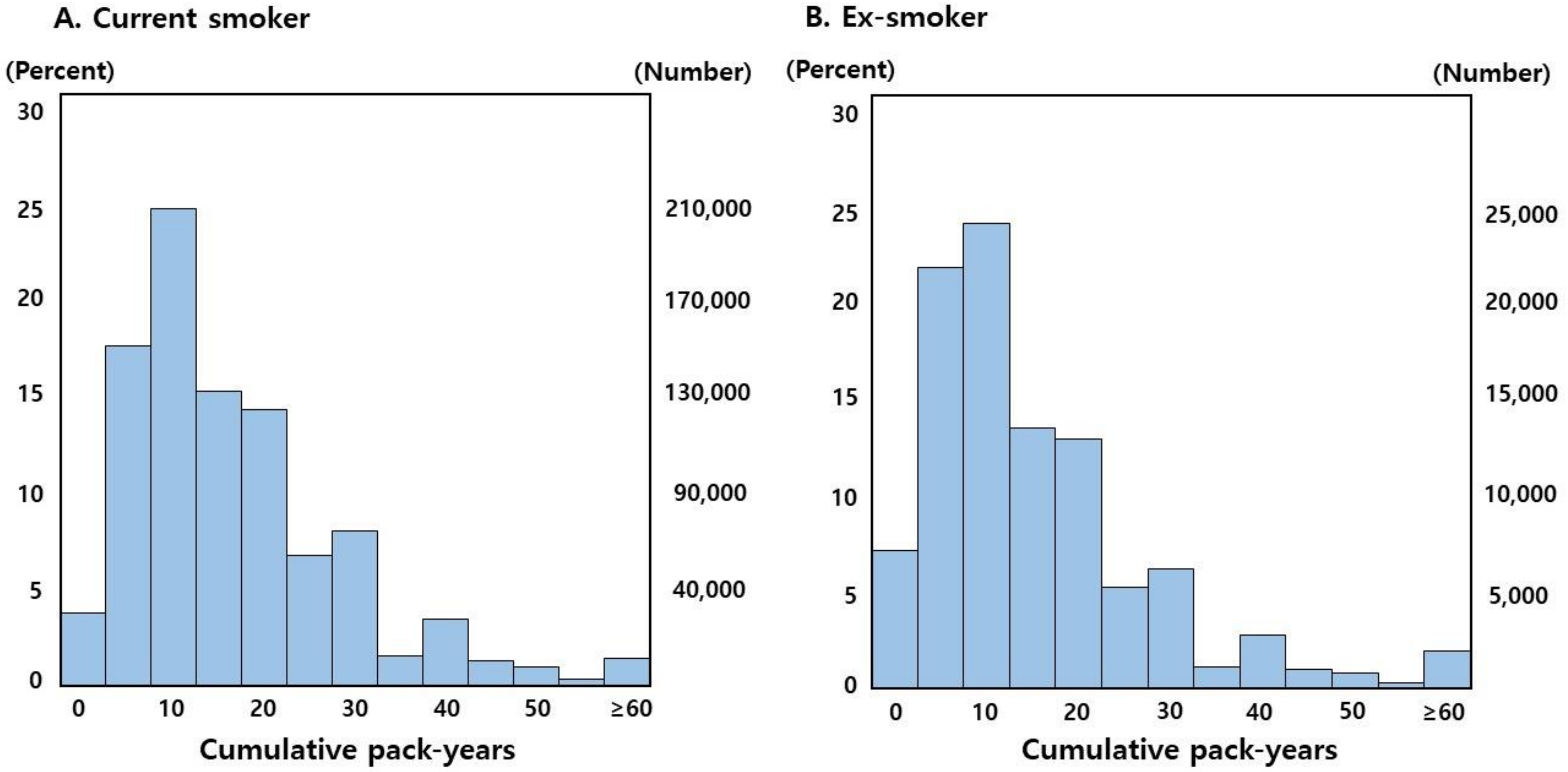

© 2024 Cho JH et al. *JAMA Network Open.*

eFigure 2. The Relationship Between Pack-Years and The Hazard Ratio of CVD in Ex-smoker and Current smoker

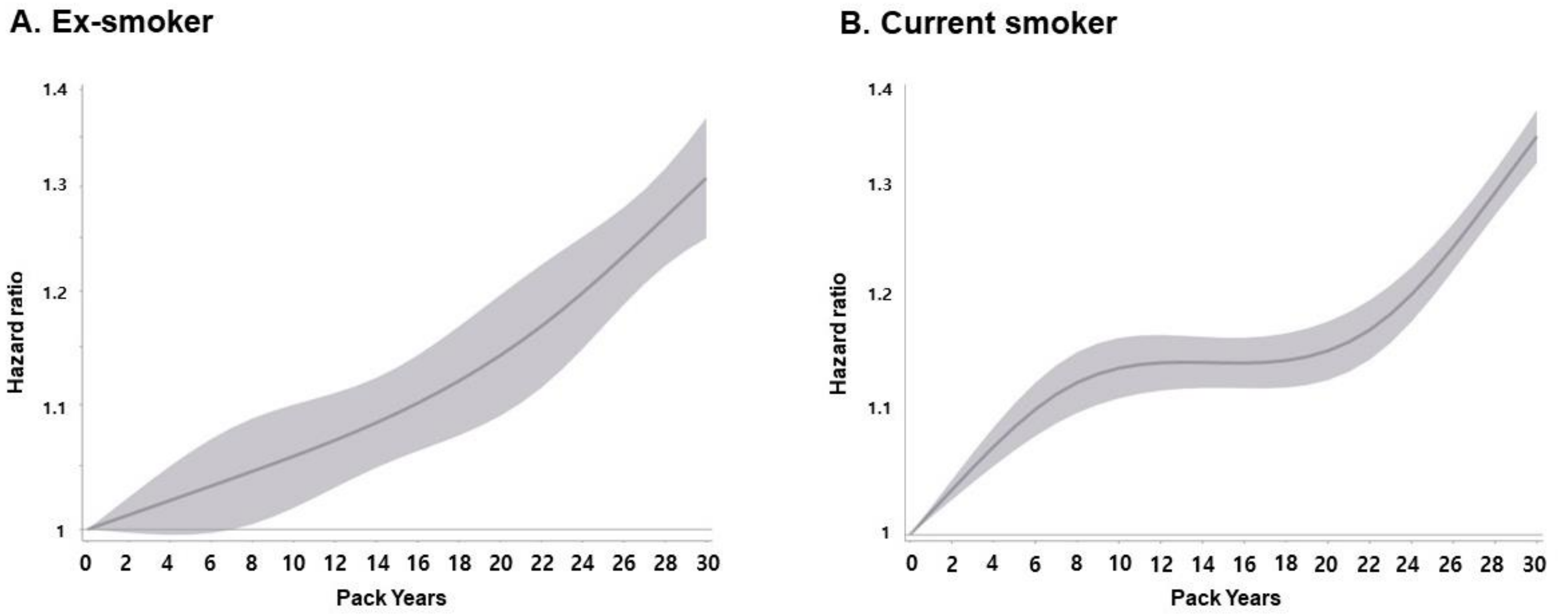

© 2024 Cho JH et al. *JAMA Network Open.*
