# Supplementary material for: Smoking Cessation and Incident Cardiovascular Disease
Source: JAMA Netw Open. 2024 Nov 1;7(11):e2442639. doi: 10.1001/jamanetworkopen.2024.42639 (PMC11530932; doi:10.1001/jamanetworkopen.2024.42639)
Supplement: Supplement 2. — Data Sharing Statement [file jamanetwopen-e2442639-s002.pdf]

Data Sharing Statement

Cho. Smoking Cessation and Incident Cardiovascular Disease. *JAMA Netw Open*. Published November 01, 2024. doi:10.1001/jamanetworkopen.2024.42639

Data
Data available: No

Additional Information
Explanation for why data not available: The data that support the findings of this study are available from the National Health Insurance Sharing Service (NHISS, https://nhiss.nhis.or.kr/) but restrictions apply to the availability of these data, which were used under license for the current study, and so are not publicly available. Data are however available from the authors upon reasonable request and with permission of the NHISS.
